# Hepatitis C virus infection in a child with autoimmune polyendocrine syndrome type 2: a case report

**DOI:** 10.1186/1752-1947-6-221

**Published:** 2012-07-27

**Authors:** Kotb Abbass Metwalley, Hekma Saad Farghaly

**Affiliations:** 1 Pediatric Endocrinology & Diabetes Unit -Department of Pediatrics, Faculty of Medicine, Assiut University, Assiut, Egypt

## Abstract

**Introduction:**

Autoimmune polyendocrine syndrome type 2 is a rare disorder. Its prevalence in western populations has been reported as 1.5 to 4.5/100,000. On the other hand, its prevalence in Egypt is unknown. It is characterized by the association of autoimmune Addison’s disease with thyroid autoimmune diseases and/or type I diabetes mellitus. Hepatitis C virus infection is an important public health issue worldwide. Egypt has the highest prevalence of hepatitis C virus infection of any country in the world. It is estimated to be 8% in urban and 25% in rural areas. We present the case of an Egyptian child with autoimmune polyendocrine syndrome type 2 associated with chronic hepatitis C infection.

**Case presentation:**

A 14-year-old Egyptian boy with type 1 diabetes mellitus was referred to our institution for an evaluation of recurrent attacks of hypoglycemia of two months duration. The initial clinical examination revealed hypotension as well as vitiligo of the skin. He had high potassium, low sodium, low cortisol, high adrenocorticotropic hormone, slightly high thyroid stimulating levels with strong positivity of anti-thyroglobulin and anti-thyroid peroxidase antibodies. The hepatitis C antibody and hepatitis C virus–polymerase chain reaction were positive. Based on these findings, a diagnosis of autoimmune polyendocrine syndrome type 2 with chronic hepatitis C was made. He was started on hydrocortisone (10mg twice daily), fludrocortisone (0.1mg twice daily) and multiple daily doses of insulin. He showed great improvement of his symptoms on the prescribed treatment.

**Conclusions:**

The importance of the early diagnosis of autoimmune polyendocrine syndrome type 2 and the possibility of its association with chronic hepatitis C infection should be considered in order to implement the proper management of such cases.

## Introduction

Autoimmune polyendocrine syndrome (APS) is a rare syndrome caused by immune-mediated destruction and involves the failure of both endocrine and non-endocrine organs. It is characterized by an insufficiency in at least two endocrine glands. Four main types of APS have been described [1]. Autoimmune polyendocrine syndrome type 2 (APS2) (Schmidt's syndrome) is defined by the coexistence of autoimmune Addison’s disease with autoimmune thyroid disease and/or type 1 diabetes mellitus (T1DM). Some of the patients may later develop other autoimmune diseases, such as vitiligo, alopecia, chronic atrophic gastritis, pernicious anemia, chronic hepatitis and celiac disease [2]. The prevalence of antibodies to hepatitis C virus (anti-HCV) among Egyptian children ranges between 3% to 9% [3]. To the best of our knowledge, the association between chronic hepatitis C and APS2 has not been reported before in the literature. We present the case of an Egyptian child who presented with APS2 associated chronic hepatitis C.

## Case presentation

A 14-year-old Egyptian boy was referred to our institution for evaluation of recurrent hypoglycemic attacks which occurred especially in the mornings during the preceding two months. He had been diagnosed with T1DM at the age of five years and was treated with insulin in a dose of 0.7 to 1U/kg since that time. Recently, he started to develop recurrent attacks of hypoglycemia for which his insulin dose was decreased to 0.3U/kg. He had lost 2.5kg of weight and developed a craving for salt. He suffered from fatigue and dizziness. He was born and resided in a small village in Upper Egypt to unrelated Egyptian parents. He was the sixth child in his family with no history of diabetes or other auto immune diseases among family members. He had frequent hospital admissions for control of blood sugar. He was born at term with a birth weight of 3550 grams. Apart from post circumcision bleeding that was done at the age of two years by a barber, there was nothing significant in his past medical history. On physical examination, his body temperature was 36.1C, pulse rate was 92/min, blood pressure was 80/45mmHg, his height was in the 10th percentile while his weight was in the 25th percentile. He had no pallor, jaundice, or hyperpigmentation of either the skin or mucous membranes. His thyroid gland was not enlarged either on inspection or palpation. He was prepubertal: pubic and axillary hair Tanner stage 1 and testicular volume 3ml. He had slight abdominal distension, his liver and spleen were not enlarged and there was no evidence of ascities. Skin examination revealed vitiligo in the form of multiple hypopigmented areas located over the chest and abdomen. Serum biochemistry on admission showed an average blood glucose level of 77mg/dL (normal range: 82 to 110mg*/*dL), hemoglobin A1c 7.5% (normal range: 3.0 to 6.1), potassium: 5.9mmol/L (normal range 3.5 to 5), sodium 121mmol/L (normal range: 130 to 145) and calcium 9.7mg/dL (normal range: 8 to 10.5). Levels of blood urea nitrogen and creatinine were within the normal range. Liver function tests revealed bilirubin 11umol/L (normal <20umol/L), alanine aminotransferase (ALT) 186IU/L (normal <50IU/L), aspartate aminotransferase (AST) 123IUL (normal <50IU/L), alkaline phosphatase 108IU/L, (normal <350IU/L), and albumin 38g/L (normal range: 35 to 45g/L). Complete blood count, serum folate and vitamin B12 levels were normal. Viral hepatitis screens (HAV IgM, HBsAg, HBsIgM and IgG, Epstein-Barr virus, cytomegalovirus and herpes simplex virus) were negative. Anti-HCV and hepatitis C virus–polymerase chain reaction (HCV–PCR) were positive, with a viral load of 88,440IU/mL. Anti-endomysial antibodies were not detected. There were no serological features of autoimmune hepatitis (anti-smooth muscle antibody, antinuclear antibody, anti-liver/kidney microsomal antibody, and anti-parietal cell antibody were negative), anti-adrenal antibodies were positive and serum immunoglobulins were normal. Hormonal assays revealed cortisol <1.0ug/dL (normal range: 6 to 24), adrenocorticotropic hormone (ACTH) > 1250pg/mL (normal range: 20 to 100), a peak cortisol level below 0.2mcg/dL after intravenous ACTH stimulation test (250mcg). Thyroid stimulating hormone (TSH) was 6.11uIU/mL (normal range: 0.4 to 5.0). Anti-thyroglobulin (Tg autoantibodies) were 217.8IU/mL (normal: <120), anti-thyroid peroxidase (TPO autoantibodies) 5176IU/mL (normal 60), and free thyroxine 1.4ng/dL (normal range: 0.7 to 2.0). The serum luteinizing hormone was 0.1mIU/ml (normal range: 2.9 to 21.7), follicle-stimulating hormone 0.2mIU/ml (normal range: 5 to 30), testosterone 0.02ng/mL (normal range: 9.0 to 40.0), prolactin 13ng/mL (normal range: 3 to 30). Insulin C-peptide was <0.50ng/mL (normal range: 1.5 to 3.5). Parathormone (PTH) was 33.4pg/mL (normal range: 2 to 72). Thyroid ultrasonography revealed heterogeneous echogenicity without any nodules, while ultrasonography of the abdomen was normal. His immunogenetic study demonstrated that human leukocyte antigen (HLA) typing was HLA-DR3. Based on combinations of 1DM, subclinical hypothyroidism due to Hashimoto’s thyroiditis, adrenal insufficiency, vililligo and HLA-DR3, a diagnosis of APS2 was made. The presence of positivity for both anti-HCV and HCV-RNA with elevated ALT and AST levels even in the absence of hepatomegaly confirmed the diagnosis of chronic hepatitis C infection. His general condition improved gradually following the administration of hydrocortisone (10mg twice daily) and fludrocortisone (0.1mg twice daily). His diabetes was controlled with intensive multiple daily insulin injections. He was referred to the regional hepatology unit for further assessment including liver biopsy and consideration of hepatitis C eradication therapy but his father preferred to delay the treatment because he was worried about the side effects of the treatment.

## Discussion

The association between Addison’s disease and other endocrinopathies was probably first described in 1886. In 1926, Schmidt presented two cases of Addison’s disease with cortical atrophy and lymphocytic infiltration of the adrenal, as well as lymphoid infiltration in the thyroid [4]. APS2 is rare, because it necessarily requires the presence of Addison’s disease. Considering that the prevalence of idiopathic Addison’s disease has been suspected to be 30 to 60 cases/million inhabitants and that about 50% to 75% of Addisonian patients may be affected by thyroid autoimmune disease and insulin dependent diabetes or both, it may be estimated that APS2 occurs in 15 to 45 cases per million inhabitants in the general population. The syndrome may occur at all ages and in both sexes but is most commonly found in middle-aged women. Inheritance is consistent with autosomal dominance with incomplete penetrance. These patients have an increased frequency of the HLA A1, B8 and DR3 haplotypes [5]. An unexpected fall in insulin requirement in patients with T1DM may be the earliest sign of adrenal failure. Our index case developed recurrent hypoglycemia attacks without any change in his treatment or physical activity. Hypoglycemic attacks are considered to be caused by enhanced insulin sensitivity secondary to glucocorticoid deficiency [6]. Hashimoto’s thyroiditis was diagnosed in our case on findings of seropositivity for Tg and TPO autoantibodies, and slightly high TSH accompanied by morphological changes seen on thyroid ultrasound [7]. Symptoms and signs of hypothyroidism and hypoadrenalism are often non-specific. Since early diagnosis and treatment of these hormonal insufficiencies are vital, it is essential to always consider an association. Adrenal insufficiency should be considered in patients with autoimmune thyroid disease or T1DM who develop non-specific illness or become seriously ill, or in diabetic patients whose insulin requirements fall inexplicably [8]. The hyperpigmention of the skin and mucous membranes was not obvious in our case. The absence or presence of hyperpigmentation in Addison’s disease is related to the duration of the illness and the time taken to stimulate the pituitary to produce ACTH. Although pigmentation is a very useful sign in Addison’s disease, its absence by no means excludes the diagnosis. It seems that only an awareness of the possibility of the disease will lead to an early diagnosis [9]. Our case showed vitiligo over the chest and abdomen which is a chronic disorder that causes patchy hypopigmentation of the skin. It occurs when the melanocytes, which are derived from the neural crest, die or are unable to function. The precise pathogenesis of vitiligo is not yet fully understood but an autoimmune etiology is the most widely accepted. It is mandatory to actively look for, and if necessary, investigate patients with vitiligo for other autoimmune diseases [10]. In Egypt, HCV infection in diabetic children is considered a health-related infection; with a prevalence of anti-HCV among diabetic children of 3.5%, it may be attributed to repeated hospitalization, insulin injections, shared insulin vials and shared spring-triggered devices for capillary blood glucose monitoring [3]. Our case was diagnosed with chronic hepatitis C. We suggest that the risk factors for contraction of hepatitis C in our case were frequent hospital admissions and circumcision by a barber which is a common practice in villages in Upper Egypt. These suggestions are supported by Kalil *et al.*[11] and Medhat *et al.*[12] in their study regarding risk factors of acquisition of hepatitis C in children in Upper Egypt. HCV seems to be the virus usually associated with the appearance of autoimmune diseases, and the relationship between chronic HCV infection and some autoimmune disease has been studied. For some of these disorders their association with HCV infection is well recognized while for others it remains probable or weak [13]. HCV infection has been associated with Type 2 diabetes mellitus; however, Chen *et al.*[14] reported T1DM one year after a blood transfusion-related HCV infection. The exact mechanisms underlying HCV-mediated T1DM still are not well understood and several investigators have proposed that cross reactive mechanisms are operating in the generation of autoimmunity in HCV infection. This hypothesis is supported by the discovery of remarkable amino acid similarities between HCV and glutamic acid decarboxylase 65 (GAD65), protein tyrosine phosphatase islet cell antigen-2 and phogrin [15]. Identification of the type and extent of autoimmune disorders in children with HCV infection is important for the following reasons: First, this awareness can result in rapid diagnosis and treatment of these diseases. Second, the existence of an autoimmune disorder in patients with HCV infection requires clinicians with good experience in the management of such cases. Third, patients presenting with these disorders can be targeted for HCV testing [13]. Our case presents three very interesting and unusual aspects. First, given that APS2 occurs most often in middle-aged women, its presentation in a boy during childhood is uncommon. In this communication, we report the case of a 14-year-old boy with APS2 with HCV infection. Second, the association of APS2 with chronic hepatitis C in our case would suggest adding APS2 to the list of autoimmune diseases apparently associated with chronic hepatitis C. Finally, hepatic dysfunction in Egyptian children with APS2 is not necessarily due to autoimmune hepatitis but can be attributed to HCV infection.

## Conclusions

As APS2 can be fatal, it is important to maintain a high index of suspicion to enable early diagnosis and start appropriate treatment. Egyptian children presenting with autoimmune disorders should be tested for HCV infection.

## Consent

Written informed consent was obtained from the patient’s next-of-kin for publication of this case report and any accompanying images. A copy of the written consent is available for review by the Editor-in-Chief of this journal.

## Abbreviations

APS2, Autoimmune polyendocrine syndrome type 2; HCV, Hepatitis C virus; T1DM, Type 1 diabetes mellitus.

## Competing interests

The authors declare that they have no competing interests.

## Authors’ contributions

KAM and HSF diagnosed, investigated, followed-up and managed the patient and drafted the manuscript. Both authors read and approved the final manuscript.

## References

[B1] Betterle C Zanchetta R Update on autoimmune polyendocrine syndromes (APS) Acta Biomed Ateneo Parmense 2003 74 9 33 12817789

[B2] Falorni A Laurenti S Santeusanio F Autoantibodies in autoimmune polyendocrine syndrome type II Endocrinol Metab Clin North Am 2002 31 369 389 10.1016/S0889-8529(01)00010-X 12092456

[B3] El-Karaksy HM Anwar G Esmat G Mansour S Sabry M Helmy H El-Hennawy A Fouad H Prevalence of hepatic abnormalities in a cohort of Egyptian children with type 1 diabetes mellitus Pediatr Diabetes 2010 11 462 470 10.1111/j.1399-5448.2009.00627.x 20042012

[B4] Ogle JW Brain disease from diabetes mellitus St George’s Hosp Rep 1866 1 17822813687

[B5] Qari FA Damnan S Schmidt’s syndrome in a Saudi family Saudi Med J 2000 21 93 95 l 11533759

[B6] McAulay V Frier BM Addison's disease in type 1 diabetes presenting with recurrent hypoglycaemia Postgrad Med J 2000 76 230 232 10.1136/pmj.76.894.230 10727569 PMC1741569

[B7] Wasniewska M Salerno M Salerno M Prospective evaluation of the natural course of idiopathic subclinical hypothyroidism in childhood and adolescence Eur J Endocrinol 2009 160 417 421 19074464 10.1530/EJE-08-0625

[B8] Tsang CC Ko GTC Wong KK Chan HS Yu AW Autoimmune polyendocrinopathy type II in a Chinese patient Hong Kong Med J 2006 5 385 387 17028360

[B9] Goodwin TJ Kind PR Bogomoletz VW Addison's disease without pigmentation Postgrad Med J 1973 49 305 308 10.1136/pgmj.49.571.305 4804454 PMC2495861

[B10] Poojary SA Vitiligo and associated autoimmune disorders: a retrospective hospital-based study in Mumbai, India Allergol Immunopathol 2011 39 356 361 10.1016/j.aller.2010.12.007 21474231

[B11] Kalil KA Farghally HS Hassanein KM Abd-Elsayed AA Hassanein FE Hepatitis C virus infection among paediatric patients attending University of Assiut Hospital, Egypt East Mediterr Health J 2010 16 356 361 20795415

[B12] Medhat A Shehata M Magder LS Mikhail N Abdel-Baki L Nafeh M Abdel-Hamid M Strickland GT Fix AD Hepatitis C in a community in Upper Egypt: risk factors for infection Am J Trop Med Hyg 2002 66 633 638 12201604 10.4269/ajtmh.2002.66.633

[B13] Jadali Z Alavian SM Autoimmune diseases co-existing with hepatitis C virus infection Iran J Allergy Asthma Immunol 2010 9 191 206 21131699

[B14] Chen LK Chou YC Tsai ST Hwang SJ Lee SD Hepatitis C virus infection related Type 1 diabetes mellitus Diabet Med 2005 22 340 343 10.1111/j.1464-5491.2005.01412.x 15717885

[B15] Bogdanos DP Rigopoulou EI Viral/self-mimicry and immunological cross-reactivity as a trigger of hepatic C virus associated autoimmune diabetes Diabetes Res Clin Pract 2007 77 155 156 10.1016/j.diabres.2006.10.012 17118481

